# Human Gut Microbiota of Healthy Individuals Show Resilience to Acute Aflatoxin B1 Exposure Despite Solvent-Driven Community Shifts

**DOI:** 10.64898/2026.10.26.760590

**Published:** 2026-10-27

**Authors:** Hannah Glesener, Nicole Dorn, Qiwen Cheng, Lee E. Voth-Gaeddert, Rosa Krajmalnik-Brown

## Abstract

Aflatoxin B1 (AFB1) is a potent mycotoxin linked to severe systemic health issues and gut microbiome disruption in in vivo models. However, it remains unclear whether AFB1 directly impairs human gut microbial composition and function or if observed dysbiosis is host-mediated. The objective of this work was to investigate the direct impact of acute AFB1 exposure on human gut microbiota from healthy individuals using anaerobic batch bioreactors. Fecal inoculum from 17 healthy adults was exposed to AFB1 concentrations ranging from 1,000 to 5,000 ppb, delivered in two solvents (ethanol and corn oil) under conditions simulating the large intestine. Metabolic activity was assessed via short-chain fatty acid (SCFA) and gas production measurements, while community structure was assessed using 16S rRNA gene amplicon sequencing. AFB1 concentrations remained stable throughout the incubations, indicating no significant microbial degradation of AFB1. Results showed that AFB1 did not significantly alter microbial SCFA output or community composition compared to solvent-only controls. Observed shifts in microbial profiles were primarily driven by the solvents used to bring AFB1 into solution rather than by the toxin. These findings demonstrate that healthy human gut microbial communities are resilient to direct, acute AFB1 exposure even at concentrations exceeding typical dietary levels. This suggests that microbiome disruption reported in vivo may be an indirect consequence of host-mediated factors, such as reactive metabolites or systemic immune responses, rather than direct toxicity by the parent compound.

**Importance:**

Aflatoxin B1 is a major global health threat, particularly in regions with high dietary exposure, where it is associated with liver cancer and childhood growth stunting. While research has linked aflatoxin exposure to changes in the gut microbiome, the mechanisms driving this “dysbiosis” are not known. This work provides evidence that the parent aflatoxin molecule does not directly affect healthy human gut microbes, even at very high concentrations. The observed resilience of these microbial communities suggests that the damage reported in living organisms is likely caused by host-specific responses to the toxin or by toxic byproducts created during liver detoxification. Furthermore, microbially driven detoxification of the parent aflatoxin molecule was not observed. Distinguishing between direct and indirect toxicity is essential for developing effective strategies and medical interventions to mitigate the health impacts of aflatoxin exposure worldwide.

## Full Text Availability

The license terms selected by the author(s) for this preprint version do not permit archiving in PMC. The full text is available from the preprint server.

